# The importance of antimicrobial resistance in medical mycology

**DOI:** 10.1038/s41467-022-32249-5

**Published:** 2022-09-12

**Authors:** Neil A. R. Gow, Carolyn Johnson, Judith Berman, Alix T. Coste, Christina A. Cuomo, David S. Perlin, Tihana Bicanic, Thomas S. Harrison, Nathan Wiederhold, Mike Bromley, Tom Chiller, Keegan Edgar

**Affiliations:** 1 MRC Centre for Medical Mycology, School of Biosciences, University of Exeter, Geoffrey Pope Building, Exeter, EX4 4QD UK; 2 Medical Research Council, Polaris House, Swindon, SN2 1FL UK; 3 Shmunis School of Biomedical and Cancer Research, George S. Wise Faculty of Life Sciences, Tel Aviv University, 418 Britannia Building, Ramat Aviv, 69978 Israel; 4 Microbiology Institute, University Hospital Lausanne, rue du Bugnon 48, 1011 Lausanne, Switzerland; 5 (CAC) Infectious Disease and Microbiome Program, Broad Institute of MIT and Harvard, Cambridge, MA 02142 USA; 6 Center for Discovery and Innovation, Hackensack Meridian health, Nutley, NJ 07110 USA; 7 Institute of Infection and Immunity, St George’s University of London, London, SW17 0RE UK; 8 Clinical Academic Group in Infection, St George’s University Hospitals NHS Foundation Trust, London, SW17 0QT UK; 9 Department of Pathology and Laboratory Medicine, University of Texas Health Science Center at San Antonio, San Antonio, TX 78229 USA; 10 Manchester Fungal Infection Group, Division of Evolution, Infection, and Genomics, Faculty of Biology, Medicine and Health, University of Manchester, CTF Building, 46 Grafton Street, Manchester, M13 9NT UK; 11 Center for Disease Control and Prevention Mycotic Disease Branch 1600 Clifton Rd, MSC-09, Atlanta, 30333 GA USA

**Keywords:** Antifungal agents, Antimicrobial resistance, Clinical microbiology, Fungi

## Abstract

Prior to the SARS-CoV-2 pandemic, antibiotic resistance was listed as the major global health care priority. Some analyses, including the O’Neill report, have predicted that deaths due to drug-resistant bacterial infections may eclipse the total number of cancer deaths by 2050. Although fungal infections remain in the shadow of public awareness, total attributable annual deaths are similar to, or exceeds, global mortalities due to malaria, tuberculosis or HIV. The impact of fungal infections has been exacerbated by the steady rise of antifungal drug resistant strains and species which reflects the widespread use of antifungals for prophylaxis and therapy, and in the case of azole resistance in *Aspergillus*, has been linked to the widespread agricultural use of antifungals. This review, based on a workshop hosted by the Medical Research Council and the University of Exeter, illuminates the problem of antifungal resistance and suggests how this growing threat might be mitigated.

## Abstract (web summary)

The impact of fungal infections on human health has been exacerbated by the rise of antifungal drug resistance. In this Review, the authors outline the problem of antifungal resistance and suggest how this growing threat might be mitigated.

## Introduction and perspective

The age of antibiotics, spanning only 80 years, is now entering a period of progressive and widespread emergence of drug-resistant organisms that threaten to bring this era to an end^1–4^. Microbial pathogens, including fungi, tend to have short generation times, plastic genomes, and the ability to adapt to natural environments that contain many potentially toxic compounds, which exert strong selective pressures. The eukaryotic biochemistry of fungi makes them particularly pernicious pathogens because of a more limited number of selective drug targets against which inhibitors can be designed that are non-toxic for human, animal, and plant hosts. Furthermore, no therapeutic vaccines or adjunct immunotherapies are available to support human health care; this necessitates reliance on a limited armoury of antifungal drug classes to treat a rising tide of fungal infections. These challenges are exacerbated by the emergence of drug resistant, tolerant, or insensitive organisms and increasing numbers of susceptible hosts. Resistant strains of fungi have been identified shortly after the introduction of new antifungal drugs and despite new antifungals in the pipeline^5^, once they are introduced clinically, we should anticipate that resistance will ultimately emerge unless mitigating strategies are deployed. Resistance is the result of genetic mutations and induced protective mechanisms. Rapid plasmid-mediated spread of resistance has not been detected in fungi (as opposed to bacteria). However, antifungal resistance and tolerance can be acquired rapidly, often by the induction of protective stress response pathways, sometimes including the acquisition of aneuploidy or other forms of copy number variation. Thus, the emergence of fungal strains and species with single or multiple drug resistance profiles poses significant challenges in the treatment of medical, veterinary and agricultural hosts^6–10^.

Of the estimated five million species of fungi, less than 100 species are frequent agents of human disease, and most deaths are due to organisms within the genera *Candida*, *Aspergillus* and *Cryptococcus*^3^. However, a cadre of new emerging pathogens are rising in clinical importance, and these include some highly drug-resistant species, including *Scopulariopsis* and *Lomentospora*^11^. Antifungal resistance. This can be a consequence of the response to patient antifungal treatment, but many human pathogenic fungi also have an environmental phase where resistance can emerge^12^. For example, antifungal resistance in *Aspergillus fumigatus* is clearly associated with environmental selection of resistance as a consequence of exposure to agricultural azoles used in crop protection^13^. Indeed, estimates suggest that one in 20 culturable isolates of this fungus isolated from the air are tebuconazole resistant^14^. Some strains of *Candida glabrata*, *Candida krusei*, most strains of *Scedosporium* and the Mucorales, and the recent emergent species *Candida auris* display reduced susceptibility to commonly used antifungals. The problems of antifungal resistance are compounded by problems of late diagnosis and consequently treatment delays. Very high levels of morbidity and mortality^1^ are associated with comorbidities, (e.g., haematological malignancies, solid organ transplantation, ICU stays, HIV, SARS-CoV-2, and influenza), rising numbers of susceptible hosts, host immune status, drug accessibility, drug tolerance, treatment with biologics and the formation of fungal biofilms. Life-threatening fungal infections also tend to be prevalent in resource-limited areas of the world with fewer health care options, including access to antifungal diagnostics and drugs. Low- and middle-income countries face additional challenges, including indiscriminate use of antifungal drugs, and limited stewardship^15,16^. Cumulatively, these factors result in hundreds of millions of serious fungal infections and between 1 and 1.5 million attributed fungal infection-related deaths per year^1,2^.

This review summarizes the conclusions of a workshop hosted by the Medical Research Council and the University of Exeter in May 2021. The workshop brought together a group of medical mycologists with diverse research interests (Supplementary Table 1) to outline the scale of the threat and the opportunities to mitigate the consequences of antifungal resistance.

### Mechanisms of antifungal resistance and tolerance

The number of fungal infections has continued to increase over the past 20 years, due, in part, to improved enumeration and identification of fungal infections by international organizations (e.g., GAFFI and SENTRY). In addition, the rate of antifungal resistance in yeasts continues to rise globally^17,18^, alongside the increased emergence of non-*albicans Candida* species^18^. The recent emergence of multidrug-resistant yeasts^19^, such as *C. auris* and *C. glabrata* is reminiscent of the situation with bacteria. Antifungal resistance in filamentous fungi, notably *A. fumigatus*^20^, has been linked to the increased use of antifungal agents, particularly azoles, both in the environment and in the clinic. Over the past 20 years, terbinafine-resistant strains of *Trichophyton* spp. have emerged in India, and 13% of these isolates are also resistant to azoles^21,22^.

*Sensu-stricto*, antifungal drug resistance, like antibacterial drug resistance, is the ability of a fungal isolate to grow well in the presence of drug concentrations that inhibit the growth of most isolates of that species. To formalize and quantify susceptibility for clinical microbiology labs, two major consortia, the Clinical & Laboratory Standards Institute (CLSI) and the European Committee on Antimicrobial Susceptibility Testing (EUCAST) have defined breakpoints as the minimal inhibitory concentration (MIC) of a drug, above which an isolate is considered resistant to clinical treatment, as well as epidemiologic cut-off values (ECVs or ECOFFs) that define the upper limit of the wild type susceptible population when breakpoints are unavailable^23^. Drug-resistant isolates are more likely to fail treatment and to cause breakthrough infections^24^.

**Fig. 1:**
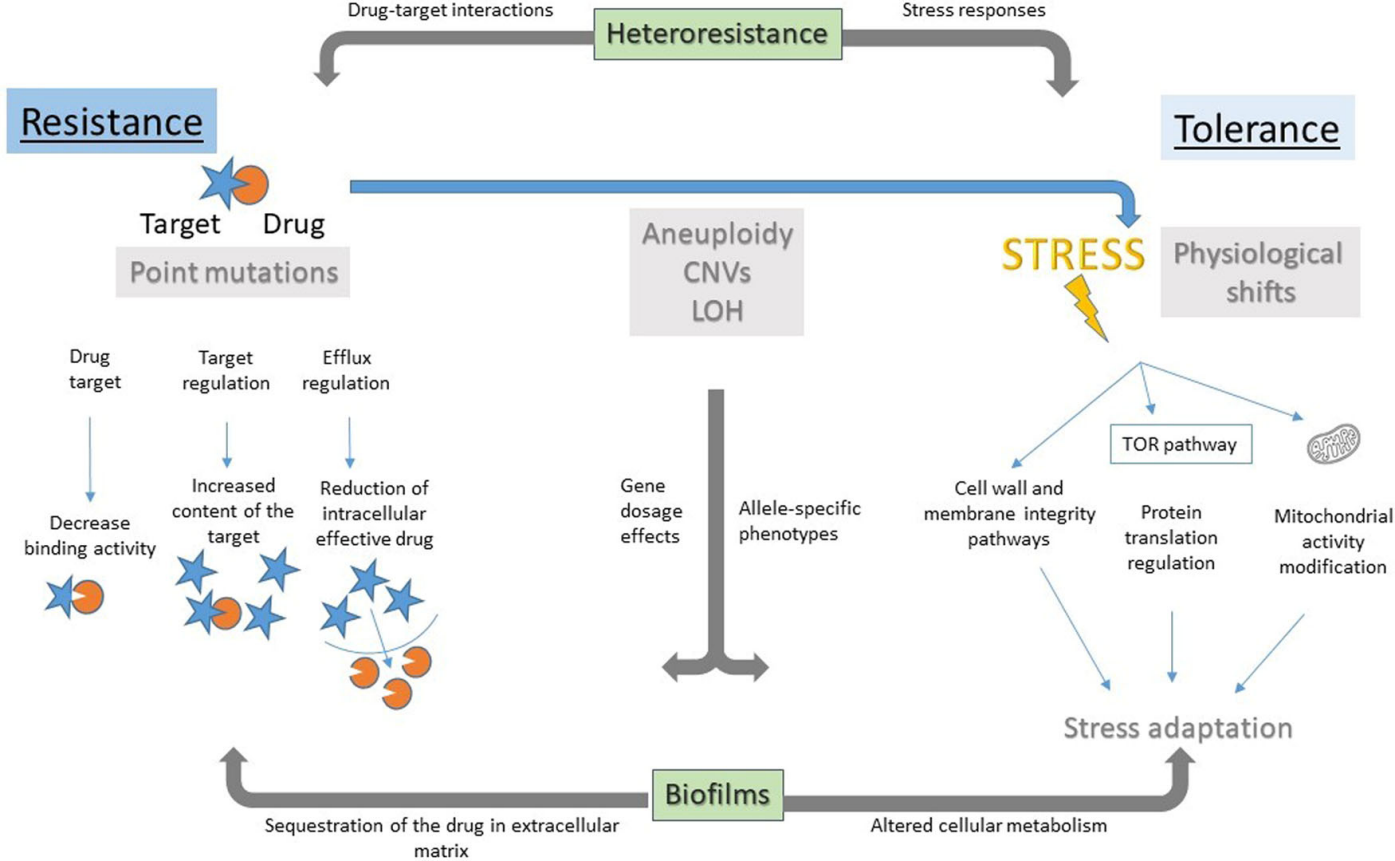
Overview of antifungal drug responses. Antifungal drug resistance (left side) is detected as elevated MIC due to direct effects on drug (orange circle) or drug target (blue star), via reduced binding affinity of the target for the drug, increased levels of the target that dilute the drug effect, or by reducing the intracellular drug concentration via drug efflux or blocked drug uptake. Antifungal drug tolerance (right side) is a physiological response to drug stress involving pathways that buffer the stress, such that some cells are able to grow, albeit slowly, in the presence of drug concentrations that are inhibitory to other cells in the population. This involves physiological shifts in: the cell wall or membrane integrity pathways (including pathways regulated by Hsp90, calcineurin, and the Crz1 transcription factor, and pathways affecting membrane lipid composition); protein translation machinery including the TOR pathway; and modifications of mitochondrial function. Loss of mitochondrial DNA in tolerant species (e.g., *C. glabrata* and *Saccharomyces cerevisiae)*, also leads to high drug efflux via Pdr1 and drug resistance, but cellular fitness is highly compromised in these ‘petite’ isolates, which are therefore not thought to be clinically relevant. Heteroresistance (across top) is a semi-stable mechanism, often due to whole chromosome aneuploidy, that can confer either resistance (increased MIC), via increased expression of a target or of efflux pumps, or tolerance (susceptible MIC but increased growth in drug) via altered stress response pathways. Biofilms (bottom) are a sessile physiological state that grows slowly and exhibits drug resistance and/or tolerance due to multiple mechanisms, including sequestration of the drug by large amounts of extracellular matrix. Aneuploidy, gene amplification, copy number variation and loss of heterozygosity (LOH) can confer resistance or tolerance, depending on the specific genes and combinations of genes that are involved.

Antifungal drug resistance is usually due to stable and heritable point mutations or insertions/deletions that directly affect the interaction of the drug with its target (Fig. 1)^20,25^. In addition to antifungal drug resistance, several more subtle drug responses that may have clinical significance have been studied primarily in yeasts. These include tolerance, heteroresistance, biofilm formation, aneuploidy, and persistence (reviewed in ref. 26).

Antifungal drug tolerance, often termed ‘trailing growth’ in clinical studies, appears as partial growth after >24 h in susceptibility assays, because tolerance is due to the slow growth of some cells in the population that eventually grow in inhibitory drug concentrations^27^. When the growing cells are re-tested, again only some progeny cells grow, implying that tolerance is a physiological or epigenetic phenomenon or that it is transient. Aneuploidy can confer resistance or tolerance as well as cross-tolerance and appears in response to a range of drugs and pathogenic yeast species^28–33^ and, like copy number variation, is maintained primarily under drug pressure. Among the specific genes that affect tolerance are genes encoding transcription factors Czf1 (ref. 34) and Gzf3 (ref. 35), an iron acquisition factor Iro1 (ref. 36) and sphingolipid biosynthesis^37^. Tolerance involves a broad range of stress response pathways, such as the cell wall and membrane integrity pathways (Hsp90, calcineurin, PKC), the TOR pathway that responds to and regulates protein translation, as well as pathways that bypass or alleviate the drug stress indirectly, such as membrane lipid biosynthesis and central metabolic pathways, where their contribution to tolerance remains to be understood (Fig. 1).

Heteroresistance to fluconazole, which has been detected in *Cryptococcus neoformans*^38^ and *C. glabrata*^39^, refers to the presence of a small subpopulation, usually <1% of the total, with intrinsic antifungal resistance, which can be selected for and become the dominant population on treatment. For example, in *C. neoformans*, a common heteroresistance mechanism is the acquisition of aneuploid chromosomes that carry genes encoding the drug target and/or efflux pump genes^38,40^, although aneuploidy does not explain all instances of heteroresistance^38,40^ (Fig. 1).

Biofilms are a physiological adaptation to surface attachment that enables survival in the face of antifungal drugs, via multiple mechanisms, including sequestration of the drug in the extracellular matrix material that is secreted in extracellular vesicles^28,41^. The physiological changes that accompany biofilm formation are transient, being lost when cells exit the biofilm state and grow as yeast. Biofilms are influenced by genetic background^29^ and can exhibit increased drug resistance and drug tolerance, although the degree of overlap between these processes remains to be explored. Finally, persistence is a concept seen in bacteria treated with bactericidal drugs, where rare (>0.1% of the population for most commonly used antibiotics^42^), metabolically quiescent cells survive by not metabolizing the cidal drug. Antifungal persistence was associated with biofilms in one study, but it has been more difficult to detect (reviewed in ref. 43) and its relevance remains controversial^44^.

Genetic background plays a major role in antifungal tolerance, with the degree of tolerance much higher in some clinical isolates than others, such as fluconazole tolerance in *Candida albicans*^35,36^. In addition, tolerance is more evident with fungistatic drugs like azoles, yet is seen with fungicidal drugs such as echinocandins in some species. It appears that *C. auris* (Box 1) is highly resistant to azoles and also exhibits high levels of azole tolerance^45–47^. In *C. glabrata*, mitochondria play a role in the appearance of tolerance to echinocandins^48^.

Tolerance or trailing growth is not quantified in diagnostic assays. However, tolerance can be measured via minor modifications of current susceptibility assays^35,36,49^. Several small-scale studies suggested that higher tolerance of invasive *C. albicans* strains contribute to treatment failures and increased patient mortality^36,50,51^. Larger clinical studies are needed to determine the degree to which tolerance plays a role in treatment failures. In addition, understanding how the complex circuitry that allows cells (or only some cells) to grow under stress conditions is an important challenge currently being explored with a range of approaches.

One major approach to studying the acquisition of resistance and tolerance is experimental evolution in the presence of inhibitory or sub-inhibitory drug concentrations. Inhibitory drug concentrations select for the rare resistant isolates, while sub-inhibitory concentrations often enable the appearance of tolerant cells^52^. The effects of variables such as the genetic background of the starting isolate and/or differences in culture conditions (in vitro and in animal models) can be evaluated by their effect on the rate of resistance mutation appearance. The evolved progeny can be analyzed using selective screens that either sequence only specific genes known to be involved in resistance (e.g., direct targets of azoles (*ERG11/CYP51A*) or echinocandins (*FKS1/FKS2*), or that use genome-wide sequencing to identify potential new resistance and tolerance mechanisms by comparing them to the progenitor strain sequence^53–55^). Mutations that affect levels of drug transporters, can also be found in highly resistant isolates^55^ and mutations in genes that affect stress response pathways are expected in tolerant isolates.

A second complementary approach is either to collect time series of isolates from single patients during a course of antifungal therapy, or to collect single isolates from large sets of patients^47,56^. Time series can be analyzed similarly to experimental evolution experiments and identified mutations can be correlated with clinical data, including changes in the application of antifungal therapies. Evaluating isolates for mutations known to confer drug resistance can establish the prevalence of specific mutations, however unstable changes such as aneuploidy, heteroresistance and physiological adaptations such as cell wall compensation changes can be missed unless drug selection is maintained^40,57^. Examining how these mutations have spread through the population by mapping them to a phylogeny of the isolates, can determine the level of stratification and number of independent resistance emergence of events^47^.

Another caution in all such studies is that when only a single isolate from a sample is examined it is likely that only the most frequent genotype will be identified. A more comprehensive view comes from analysis of many isolates from the same patient sample and repeated sampling over time^58,59^. This can reveal the variation within a host and the frequency with which mutations are maintained or lost, such as when the drug treatment is altered. Because the frequency of cells that carry a mutation conferring drug resistance may change over time, such studies can evaluate the degree of variation between the isolates collected from a single patient sample.

Genome-wide sequencing of large sample sizes allows the evaluation of mutation frequencies that correlate with resistance. Examining the sequence of genes linked to resistance in clinical isolates that exhibit resistance requires an understanding of how the mutation may affect cellular properties linked to drug sensitivity. Identifying variants systematically associated with resistance or tolerance, such as by genome-wide association studies, has the potential to identify new mechanisms of resistance. Genome sequencing can also detect correlations across studies, between the types of genome changes that arise in vitro versus those that arise in clinical samples^55^, which can strengthen confidence in the clinical relevance of those gene alleles, copy number variations, or aneuploidies.

Evaluating the genes essential for growth in the presence of drugs can identify new mechanisms important for resistance. Several screens have identified mutants that cannot grow in the presence of an antifungal drug—these include screens of large-scale gene deletions^60^, conditionally repressed strains^61^ and in vivo transposon libraries^62^. Recent studies have sought to infer gene essentiality comprehensively, under any condition or in the presence of drug, by combining data from in vivo screening, genetic interactions, and gene expression using machine learning^62^ or neural network algorithms^61^. Carrying out such experiments with large-scale mutant collections can provide a comprehensive catalogue of resistance mutations and an estimate of the rate at which resistance arises.

Once candidate mutations are identified, either via evolution in vitro, in animal models, or in clinical isolates, reverse genetic functional tests can introduce the specific change into a sensitive isolate and/or correct the change in the resistant isolate, and then analyze the relevant drug responses^55^. For copy number and aneuploid mutants, deleting or overexpressing those genes hypothesized to be causative can support or refute the hypothesis.

Multi-disciplinary approaches are needed to underpin development of clinical strategies to mitigate antifungal resistance. These include using experimental evolution in vitro and in more clinically relevant infection models to study ex vivo micro-evolution in serial clinical isolates from relevant infection sites. These studies would be further enhanced by incorporating other factors contributing to clinical failure such as drug exposure and treatment response biomarkers.

Box 1 The urgent threat of *Candida auris* drug resistance *Candida auris* represents a major threat to global health as resistance to multiple classes of antifungal drugs is common. The pathogen has a unique ability to colonize human skin and mucosa and persist on surfaces in hospital and nursing home environments, including hands of healthcare workers, bed rails, medical equipment, and other surfaces, causing difficult-to-eradicate outbreaks^74^. Whilst a rare cause of candidaemia in most centres in Europe and the USA, a much higher prevalence has been reported from other parts of the world. For example, *C. auris* was reported as the leading cause of candidaemia (40%) at a North Indian tertiary hospital ICU, and accounted for 14% of all candidaemias reported in South Africa in 2016-2017, and 38% of all candidaemias at a single centre in Kenya in 2010–2016 (refs. 128, 129). Risk factors for *C. auris* candidaemia include prolonged hospital and ICU stay, prior antifungal treatment, older age and having a central venous catheter in situ^130–132^.

Most *C. auris* isolates are resistant to azoles, roughly half also display lower sensitivity to the polyene amphotericin B^76^, and 41% of isolates studied are multidrug resistant. Resistance or tolerance to echinocandins, the preferred treatment, can emerge on treatment, and pan-resistance to all three drug classes has been reported. *C. auris* is genetically diverged from more commonly observed *Candida* species, and the closest related species in the *Candida haemulonii* complex also display high rates of resistance. Studies of the genetic basis of resistance have identified high frequency mutations in drug targets (*ERG11* and *FKS1*) as well as in the Tac1B and Mrr1 transcription factors^16^ that control drug transporter expression that increase resistance to azoles and echinocandins, which vary in type and frequency between the four major genetic clades of *C. auris*^47,48^. By contrast, the mechanism of amphotericin resistance is largely unknown, aside from a report linking unusually high resistance levels to a loss of function mutation in the ERG6 protein involved in ergosterol biosynthesis^129^. *C. auris* is also highly adaptive to a wide variety of stressors including drug pressure and displays genetic diversity (manifest by a variety of karyotypes) and ability to develop drug tolerance during therapy, which pave the way for development of resistance^132,133^. New drugs such as those in late-stage clinical development that represent novel targets are needed to thwart this daunting multidrug-resistant pathogen.

### Clinical consequences of antifungal resistance

**Fig. 2:**
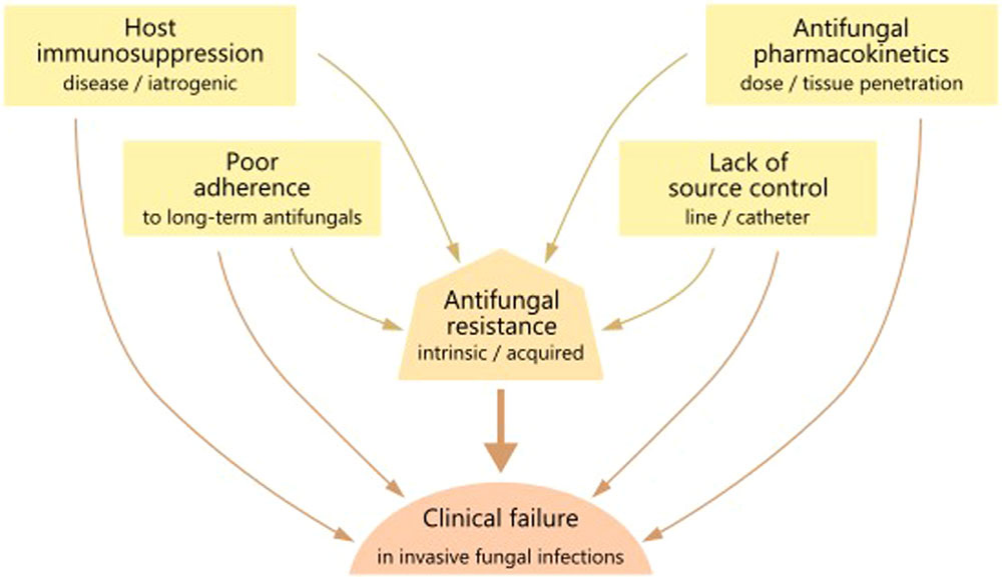
Factors mediating the contribution of antifungal resistance to clinical failure. All of the factors contributing to clinical failure in invasive fungal infection are also drivers of antifungal resistance.

*Aspergillus*, *Cryptococcus* and *Candida* spp. are the dominant human fungal pathogens globally, causing invasive infections of the lung, brain, and bloodstream, respectively. Repeated use of antifungals in at-risk groups, empiric, or targeted therapy of mucosal or invasive fungal infections, as well as the widespread use of azoles in agriculture, have altered the landscape of fungal species displaying resistance to one or more classes of antifungals. Most concerning is the triazole-resistant mould *A. fumigatus* and multidrug-resistant yeast species such as *C. glabrata* and *C. auris*^63^. Antifungal resistance threatens the limited antifungal armamentarium and affects clinical outcomes by delaying mycological clearance, and increasing breakthrough infections, relapse, and excess mortality. Intrinsic or acquired antifungal resistance are factors contributing to clinical failure in human infection (Fig. 2). Resistance is also potentiated by a number of factors such as: host immunosuppression (resulting in persistence or delayed clearance of infection); suboptimal antifungal pharmacokinetics (due to low oral bioavailability, lack of therapeutic drug monitoring, poor long-term treatment adherence together with inadequate antifungal drug dose, duration and/or penetration to the site of infection); and lack of source control with fungal persistence in difficult-to-reach niches such as deep-seated abscesses and device-associated biofilms^64–66^ (Fig. 2).

*Aspergillus* spp. triazole resistance has been described in both environmental and clinical isolates from patients with pulmonary aspergillosis. Agricultural fungicides and long-term triazole treatment in individuals with chronic lung disease select for triazole resistance. Resistance prevalence varies by geographic region and patient population, with reported ranges between 1 and 10%, with some ICU cohorts from the Netherlands having >25% resistant isolates^67,68^. A 2011–2015 Dutch retrospective cohort study on cultured *Aspergillus* isolates from ICU and non-ICU patients reported a 19% frequency of azole resistance, with higher 6-week mortality for triazole-resistant invasive aspergillosis compared to triazole-susceptible infection treated with the first-line agent voriconazole^69^. European guidelines advocate using liposomal amphotericin B or voriconazole-echinocandin combination therapy where rates of triazole resistance exceed 10%^70^: however, routine surveillance is hampered by challenges in obtaining respiratory specimens from vulnerable patient groups and limited access to phenotypic (MIC) or genotypic (*Cyp51A*) azole susceptibility testing in hospital laboratories. Increasing exposure, diagnostic dilemmas and resultant azole exposure for aspergillosis associated with influenza and COVID-19 infections represent further drivers for resistance emergence^71^ and call for adequately powered trials on the efficacy of combination therapy against invasive aspergillosis in a broad range of patient populations.

In HIV-associated cryptococcal meningitis, intrinsic heteroresistance to fluconazole in clinical isolates of *C. neoformans* and *Cryptococcus gattii* is associated with reduced fungal clearance and relapse due to secondary fluconazole resistance, even when used at the high currently recommended doses of 1200 mg/d^72,73^. Combination therapy of fluconazole with flucytosine eliminates the emergence of resistant subpopulations, improving fungal clearance compared to fluconazole alone^40^.

The predominant cause of mucosal and invasive candidiasis, *C. albicans*, is intrinsically sensitive to antifungals: however, acquired resistance can evolve with prolonged or repeated exposures to antifungals (e.g., recurrent oral, oesophageal, or vulvovaginal candidiasis). Due to factors described in Fig. 2, a robust correlation between fluconazole MIC and clinical success in candidiasis is challenging to establish. A review of 1295 patient-episode-isolate events (692 mucosal and 603 invasive candidiasis) from 12 published clinical studies demonstrated an overall success rate of 85% for those episodes in which the fluconazole MIC was ≤8 μg/ml (sensitive), 67% for with MIC 16 to 32 μg/ml (sensitive, dose-dependent), and 42% for with resistant isolates (MIC ≥ 64 μg/ml)^74^. *C. glabrata* is another prominent cause of mucosal and bloodstream infections. This species has intrinsic heteroresistance to azoles and evolves stable resistance to both azoles and echinocandins following drug exposure, generating MDR isolates refractory to conventional therapy^75^. The SENTRY Antifungal Surveillance Programme reported an increase in worldwide prevalence of fluconazole-resistant *C. glabrata* from 8.6% to 10.1% from 1997–2014 and echinocandin resistance ranging between 1.7−3.5%; of concern, 5.5–7.6% *C. glabrata* isolates were resistant to both echinocandins and azoles^76^. In some tertiary care centres in the US, echinocandin resistance exceeds 13%, with elevated echinocandin MICs and the presence of FKS mutations predicted by prior echinocandin exposure and associated with clinical failure and 30-day mortality^77^. The gastrointestinal tract is largely considered the main reservoir for selection of drug resistant *C. glabrata*. These major fungal pathogens are now joined by *C. auris* as a major AMR concern. This is a newly emerged fungal pathogen classified as an urgent global threat by public health agencies due to its high transmissibility and multidrug resistance to azoles, polyenes, and sometimes echinocandins^78–80^ (Box 1).

### Translational pipeline and strategies to reduce and mitigate antifungal resistance

#### Stewardship

Historically, antifungal drugs have been used in many patients without fungal infections through prophylactic and empiric treatment strategies. This problem is exacerbated by the poor sensitivity of traditional culture-based diagnostics, and the potentially fatal consequences of treatment delay in vulnerable patient groups, such as those with haematological malignancies^64,65^. Such broad use has inevitably increased selection for secondary drug resistance, and breakthrough infections by resistant species. For fungi, notably *Candida* spp. that can be transmitted from patient to patient, population level resistance may also rise and spread^81,82^. Further development and wider adoption of stewardship programmes is needed to ensure that prescribing follows evidence-based guidelines, and future research may be guided by the identification of biomarkers of drug resistance^83,84^. A body of literature attests to the fact that stewardship programmes can reduce inappropriate prescribing, and thus reduce selective pressure, without adversely affecting clinical outcomes – although such studies may often be insufficiently powered to detect changes in clinical endpoints compared with changes in drug use or expenditure^85,86^. Reporting systems and target setting have been used to monitor and promote best prescribing practice for antibacterials and could be adapted for antifungals to improve infection control including improving hospital hygiene, contact precautions based on screening for patients colonised with drug resistant organisms, and interventions to restrict the overuse antimicrobials^87^.

**Fig. 3:**
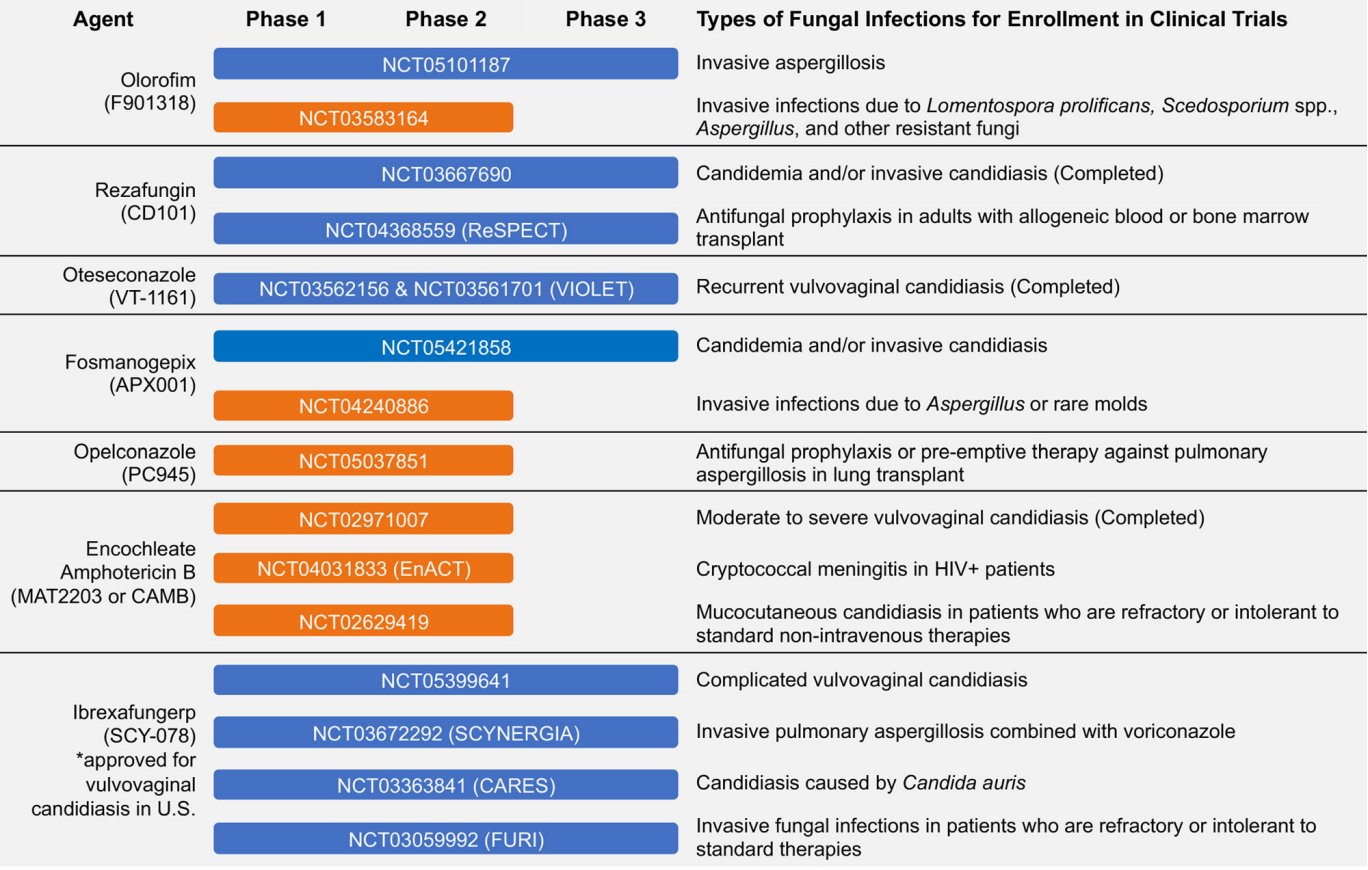
New antifungal drugs in the clinical pipeline. Antifungals that are currently in phase 2 or 3 clinical trials for the treatment or prophylaxis of fungal infections. The antifungal names as well as other identifiers are provided, along with the clinical trial number and phase, and the types of fungal infections for enrolment. Information was obtained from ClinicalTrials.gov, a database of publicly and privately funded clinical studies (accessed June 27, 2022).

#### Improved diagnostics

Fortunately, advances in diagnostics have enabled a shift towards more targeted pre-emptive treatment. PCR and immunoassay-based diagnostics for fungal invasive disease have become the mainstay for most well-resourced clinical diagnostics laboratories, and can deliver on-site results in under 12 h either at point of care or from minimally processed clinical samples^88–90^, with comparable or improved sensitivity compared with culture^91,92^. Pan-fungal β-D glucan assays are widely used to screen for fungal infections from clinical specimens, alongside more species-specific diagnostics such as the *Aspergillus* Platelia assay (Galactomannan; Bio-Rad) and several PCR-based assays. Unfortunately, these assays are often only available in reference or specialist centres, which can extend turnaround times leading to delayed treatment. Greater diagnostic mycology laboratory capacity is needed, as well as near-bedside tests such as the cryptococcal and *Aspergillus* lateral flow assays^93,94^.

Few current commercial assays can specifically identify intrinsically resistant species or detect strains that have acquired resistance^95^. Thus, MIC determination following culture continues to be the gold standard for resistance detection, although this time-consuming diagnosis is usually obtained too late to influence clinical outcome^96^.

A recently described pyrosequencing-based diagnostic directly screens respiratory samples for mutations associated with azole resistance in *CYP51A* of *A. fumigatus*. This assay has the advantage of rapidly detecting resistance even where culturing has not been possible^96^, allowing a rapid switching of therapy, when a signal is detected. However, because only 50% of azole-resistant clinical isolates have SNPs in *CYP51A*, the negative predictive value of this test is nominal.

The implementation of next-generation sequencing technologies in fungal diagnostics has the potential to provide further diagnostic granularity and to enable the detection and differentiation of multiple fungal species from a single sample. DNA metabarcoding using genomic targets such as *ITS1* (ref. 95) can identify atypical pathogens within 12 h of acquiring a sample. While the cost and technical expertise required for metabarcoding diagnostics is not prohibitive for labs with molecular diagnostic experience, several technical hurdles remain to be overcome. These include identifying short genomic targets with the diagnostic potential to distinguish sub-species of pathogenic fungi, and the availability of DNA databases with suitable diversity accurate curation.

Metagenomic diagnostics, which involve sequencing all DNA from a sample without the need for amplification, is revolutionising resistance detection for tuberculosis^97^. However, implementing metagenomic diagnostics for invasive fungal diseases is currently limited by the increased cost of sequencing large fungal genomes, low coverage of the fungal genome that may limit precision resistance diagnostic assessments to be made, and by the degree to which we understand the molecular mechanisms that contribute to antifungal resistance.

Further work, and strategic trials are needed to develop and integrate new molecular diagnostics that include detection of resistance into novel management pathways. Such pathways could enable rapid targeted therapy and improved clinical outcomes for patients with fungal infections, as well as the safe discontinuation of antifungal treatment for patients without evidence of fungal infections. In resource-limited settings, laboratory mycology based on low-cost culture-based assays and near-bedside tests is paramount.

#### New antifungal drugs

Several new antifungals are currently in either pre-clinical or clinical development (Fig. 3). Some new agents are within established drug classes that may offer advantages to currently available agents. These include: (1) rezafungin, an echinocandin with a long half-life that may allow for less frequent intravenous administration; (2) encochleated amphotericin B, which is administered orally; (3) oteseconazole, a tetrazole whose structure may be more specific for fungal lanosterol 14α-demethylase than human cytochrome P450 enzymes, thus leading to fewer drug-drug interactions, and (4) opelconazole, a triazole specifically designed for inhaled delivery^98–100^.

There are also several therapeutic candidates in development that represent new antifungal classes with novel mechanisms of action. Ibrexafungerp, which received U.S. FDA approval in 2021, is the first member of the triterpenoid class, which, like echinocandins, inhibits the synthesis of 1,3-β-D-glucans, and can be administered orally^98–100^. Two other candidates that are currently in clinical trials are olorofim and fosmanogepix. Olorofim is the first member of a novel class of antifungals, the orotomides, which target fungal pyrimidine synthesis through inhibition of the enzyme dihydroorotate dehydrogenase, thus limiting the formation of uridine-5'-monophosphate (UMP) a key precursor of DNA and RNA synthesis^101^. Olorofim is unique in that it has activity against many pathogenic moulds, including those that have reduced susceptibility to other antifungals (e.g., *Scedosporium* spp., *Microascus/Scopulariopsis*) or are pan-resistant (e.g., *Lomentospora prolificans*). However, olorofim lacks activity against yeasts, as well as the Mucorales. Manogepix is the active component of fosmanogepix, a prodrug that is rapidly converted to the active moiety by systemic phosphatases following administration^102^. Manogepix targets glycosylphosphatidylinositol (GPI)-anchored protein maturation by inhibiting the fungal inositol acyltransferase enzyme GWT1, which is responsible for trafficking and anchoring mannoproteins to the fungal cell membrane and cell wall^103^. Manogepix has broad-spectrum activity against yeasts and moulds, including strains with acquired resistance to different antifungals, including azole-resistant *A. fumigatus*, *Fusarium* sp., *Scedosporium* sp., *Lomentospora* sp., *C. glabrata* and *C. auris*^102,104^. Thus, both olorofim and manogepix may offer hope against resistant pathogens. Nonetheless, it must be noted that resistance has been observed in vitro with exposure to each of these therapeutic candidates^105–107^.

#### Combination therapy

The extension of our antifungal armamentarium could open the door to combination therapy strategies analogous to those that have proven so successful in the treatment of a wide variety of bacterial, viral, and parasitic infections. The clearest rationale is to suppress the development of resistance to a single agent, especially when the genetic barrier to resistance is low, the infectious organism load high, and treatment duration long. In addition, combinations may act additively or synergistically to increase microbial killing, potentially allowing dose reductions of one or other agent if toxicity is dose-limiting. In cryptococcal meningitis, the addition of flucytosine to fluconazole has been demonstrated to prevent the selection of hetero-resistant colonies that otherwise leads to treatment failure^40^, and combinations of flucytosine with fluconazole and with amphotericin B accelerate the rate of clearance of infection and reduce mortality^108–111^ This is an additional benefit to the original rationale for the use of this combination being therapy which was to decrease toxicity by lowering the drug dosage^108^.

Further work is needed to efficiently test combinations of existing, repurposed, and new agents against other systemic and chronic fungal infections, including candidemia, invasive aspergillosis and chronic pulmonary aspergillosis, given increasing resistance to and high attributable mortalities despite currently recommended monotherapies. Studies may be clinical and post-licensing as with cryptococcal combinations, where phase-II early fungicidal activity studies were crucial to efficiently select combinations for phase III trials^108,110,111^. Alternatively, studies may be initiated earlier in new drug development, based on careful PK-PD studies in animal models, and driven by industry or academia. Care should be taken that combinations are not used without good evidence of efficacy - especially given that some combinations maybe antagonistic, at least in vitro^112^. Within industry, the priority must be to obtain licensure, usually with use in monotherapy, although in tuberculosis and HIV there is strong precedent for the licensing of treatments in combination^113^, which provides a possible additional pathway for new drugs including any selected from the start for synergies with current agents. Development of penicillin/penicillinase inhibitor combinations for bacterial infections provides a specific example of such an approach that has proved to be of enduring value in the clinic^114^.

International collaboration across multiple sites, co-funding mechanisms, and, where possible, simplification of trial procedures and data collection, could facilitate adequately powered combination studies. The importance of this point can be exemplified by the results of a previous clinical trial of an azole-echinocandin combination for invasive aspergillosis: the trial was underpowered (70% power to detect a 60% reduction in mortality), so that although mortality was 30% lower with the combination, the benefit did not reach the conventional level of statistical significance^115^.

### Policy, communications, and advocacy

Similar to other drug-resistant diseases, the scope of potential policy work for antifungal resistance is large. There are many diverse systems that impact the development and proliferation of antifungal-resistant pathogens, including agriculture, health care, surveillance, diagnostic testing, and drug development. Each of these systems, including those discussed earlier, affect resistant fungi in unique ways, as outlined in Table 1.

**Table 1:** Systems that have an impact on antifungal drug resistance

System	Broad policy goals
Agriculture	Prevention of resistance developed through commercial fungicide use, especially azole-resistant *Aspergillus fumigatus*
Health care	Prevention of resistance developed through inappropriate prescription and use of antifungals, especially azoles Continue development of appropriate treatment plans to reduce the burden of disease Reduce transmission of resistant fungi, particularly *Candida auris*, in the health care setting
Surveillance	Improve understanding of disease epidemiology
Diagnostic testing	Increase knowledge of disease epidemiology and inform prescribing patterns, with earlier tailoring of therapy. An example is the use of cryptococcal antigen lateral flow assays (CrAg LFA)^123,124^ to estimate disease burden and facilitate early treatment in Sub-Saharan Africa.
Drug development	Ensure the continued availability of effective antifungals for all pathogenic fungi to reduce the burden of disease. An example is WHO guideline and Unitaid access support of the use of flucytosine and liposomal amphotericin B for the treatment of Cryptococcosis in Africa^126,127^

This diversity presents both challenges and advantages to policy development and communication. Developing clear and convincing evidence-supported messages to encourage action in each of these systems requires significant time, effort, and relationship-building. However, this also represents a great opportunity for action. There are numerous pathways by which effective policy can make an impact, even if there is not universal concurrence; the development of new drugs will reduce the burden of disease, even if surveillance efforts remain underfunded. As a result, effective communications employ strategies that appeal to disparate groups and capitalize on existing communication and policy channels targeted toward these groups. For example, the US Environmental Protection Agency recently added a list of cleaning products effective against *C. auris*, adding to existing lists of cleaning products designed to reduce healthcare-acquired infections^116^.

Fungal disease is not typically considered a top priority when considering funding, research, and health policy—indeed typically only 3% of infectious disease research budgets support medical mycology^3^. This may change as more data are collected demonstrating the burden of these diseases. However, encouraging the inclusion of fungi in high-profile issues may be critical to spreading awareness. For example, antibiotic resistance is an issue of concern, and many policymakers may be more likely to consider the issue of antifungal resistance when the issues are packaged together, as in the US Centers for Disease Control and Prevention’s (CDC) ‘Antibiotic Resistance Threats in the United States’ report^117^.

For example, the US CDC included *C. auris* as an “urgent threat” and *Aspergillus fumigatus* on the “watch list” of antibiotic-resistant threats. This led to these pathogens inclusion in policy groups, such as the Presidential Advisory Council for Combating Antibiotic-Resistant Bacteria (despite being fungal pathogens). In turn, both pathogens were included in the Antibiotic-Resistant Lab Network and received dedicated funding because of their classification. While these pathogens might not have garnered the same interest when communicated separately, they earned more awareness and resources because they were presented with other serious threats. Similar examples globally can be seen in the inclusion of fungal disease in initiatives such as the WHO’s Global Antimicrobial Resistance Surveillance System (GLASS)^118^.

Unlike other pathogens for which the resistance pathways, risk factors, epidemiology, diagnostic practices, and treatment are well-defined and well-known, fungal pathogens pose unique challenges to communicators and advocates. Recently the WHO has extended an invitation to participate in a survey to create a priority list of fungal pathogens^119^. Policymakers may understandably feel some hesitancy in implementing policies given this uncertainty. When communicating policy concerns and recommendations, acknowledging what remains unknown and focusing effort where there is convincing evidence that policy changes will improve health is critical. This is especially true where there are potentially significant costs associated with a policy, such as changes in the commercial use of azole fungicides, which is increasingly implicated in the development of resistant A*spergillus*^120,121^.

Data do not tell the whole story, however, and patient or patient advocacy groups can help target audiences humanize the impact of fungal diseases. While data are useful to understand the burden across populations and create policies to reduce that burden, the true impact of these diseases can be lost in the numbers and among competing priorities. Patient stories can help engage policymakers, the public, and researchers alike. These stories are also able to be used in many formats, as verbal testimony, written letter, or even on social media^122^.

As progress is being made within countries, we cannot forget the importance of international collaboration. Many countries do not have dedicated public health staff to address fungal disease, but nearly every country is, or will soon be, impacted in some way by antifungal-resistant pathogens as spread continues. Drug-resistant fungal infections are becoming more common across Europe^123^. *C. auris* has rapidly spread throughout the globe, including in many low- and middle-income countries without existing resources to combat these threats. Moreover, many cases emerging in previously naïve countries are linked to travel, including the sentinel case in the western US^124^.

Despite the caveats inherent to health communication of any variety, it is crucial to remember that prevention works. Prevention is the most cost-effective solution we have to combat resistant fungal infections, and policy and communications are key tools to improve prevention activities. Given limited funding opportunities, public health efforts supported by science are going to be a beneficial investment; it is just a matter of teaching others that they will be as well.

## Conclusions

The impact of fungal disease, potentiated by drug resistant infections, has become an urgent health priority, but innovation and progress have been limited by capacity in both discovery and translational research sectors. Without enhanced visibility of mycology to all stakeholders, including funders, researchers, industry, patients, and the public, it will be difficult to incentivize the development of capacity in this area and to catalyse interdisciplinary working to encourage step changes in therapeutic and diagnostic opportunities for the treatment of fungal infections. Dispersed specialist communities can achieve greater impact and effective advocacy through global coordination and integration of their work with allied fields of public health and infection biology to make inroads into public and private sector investment.

It is clear that the value of development of new broad-spectrum therapeutic options that are already in the pipeline would be augmented by improved diagnostics and greater understanding of the conditions and mechanisms that promote resistance and tolerance.

This position paper outlines significant progress in these respects, yet the global burden of serious fungal infection remains high, and trends continue upwards. Investment now is needed to reverse these trends and to adopt an integrated One Health approach encompassing environmental, clinical, agricultural, and social perspectives that is reviewed by GAFFI^125^. Without this investment it is possible or probable that drug resistant fungal infections will increasingly compromise successful treatment of mycotic disease.

## Supplementary information

Supplementary Information
