# Supplementary material for: The importance of antimicrobial resistance in medical mycology
Source: Nat Commun. 2022 Sep 12;13:5352. doi: 10.1038/s41467-022-32249-5 (PMC9466305; doi:10.1038/s41467-022-32249-5)
Supplement: Supplementary file 1 — Supplementary Information [file 41467_2022_32249_MOESM1_ESM.pdf]

Supplementary Table 1: List of participants in the workshop 'Drivers of antifungal resistance: strategic perspectives and priorities', hosted by the Medical Research Council and the University of Exeter on May 4th 2021.

Ana Alastruey-Izquierdo (Instituto de Salud Carlos III Campus de Majadahonda, ES)
Jorge Amich Elias (University of Manchester, UK)
Darius Armstrong-James (Imperial College, UK)
Padmapriya Banada (Rutgers University, US)
Liz Ballou (University of Exeter, UK)
Elizabeth Berkow (Center for Disease Control, US)
Judith Berman (Tel-Aviv University, IL)
Tihana Bicanic (University of Exeter, UK and St George's University of London, UK)
Elaine Bignell (University of Exeter, UK)
Margherita Bertuzzi (University of Manchester, UK)
Jessica Boname (Medical Research Council, UK)
Andy Borman (Public Health England, UK)
Paul Bowyer (University of Manchester, UK)
Axel Brakhage (Hans Knöll Institute, DE)
Michael Bromley (University of Manchester, UK)
Al Brown (University of Exeter, UK)
Emily Brown (Medical Research Council, UK)
Gordon Brown (University of Exeter, UK)
Sascha Brunke (Hans Knöll Institute, DE)
Alessia Buscaino (University of Kent, UK)
Arturo Casadevall (Johns Hopkins University, US)
Tom Chiller (Center for Disease Control, US)
Anuradha Chowdhary (University of Delhi, IN)
Arnaldo Colombo (Universidade Federal de São Paulo, BR)
Alix T Coste (University Hospital Lausanne, CH)
Christina Cuomo (Broad Institute, US)
Dame Sally Davies (UK Special Envoy on Antimicrobial Resistance, UK)
David Denning (University of Manchester, UK)
Dennis Dixon (National Institute of Allergy and Infectious Diseases, US)
Iuliana Ene (Brown University, US, Institut Pasteur, FR)
Keegan Edgar (Center for Disease Control, US)
Matthew Fisher (Imperial College, UK)
Will Gaze (University of Exeter, UK)
Gustavo Goldman (Universidade de São Paulo, BR)
Nelesh Govender (University of Witwatersrand, SA)
Campbell Gourlay (University of Kent, UK)
Neil Gow (University of Exeter, UK)
Sarah Gurr (University of Exeter, UK)
Ivana Gudelj (University of Exeter, UK)
Thomas Harrison (University of Exeter, UK and St George's University of London, UK)
Alwyn Hart (Environment Agency, UK)
Charlotte Hall (Medical Research Council, UK)
Joseph Heitman (Duke University, US)

Carolyn Johnson (Medical Research Council, UK)
Mike Hodges (Amplyx, US)
Martin Hoenigl (UC San Diego, US)
William Hope (University of Liverpool, UK)
Michael Hoy (Duke University, US)
Bernhard Hube (Hans Knöll Institute, DE)
Joseph Heitman (Duke University, US)
Joanna Jenkinson (Medical Research Council, UK)
Elizabeth Johnson (Public Health England, UK)
Katy Kettleborough (LifeArc, UK)
Anna Kinsey (Medical Research Council, UK)
Dimitrios P. Kontoyiannis (University of Texas, US)
Milena Kordalewska (Center for Discovery and Innovation, Hackensack Meridian health, US)
Damian Krysan (University of Iowa, US)
Oliver Kurzai (University of Würzburg, DE)
Baoying Liu (National Institute of Allergy and Infectious Diseases, US)
Shawn Lockhart (Center for Disease Control, US)
José Lopez-Ribot (University of Texas, US)
Dona Love (National Institute of Allergy and Infectious Diseases, US)
Johan Maertens (KU Leuven, BE)
Kieren Marr (Johns Hopkins University, US)
Kerry McLaughlin (Medical Research Council, UK)
Scott Moye-Rowley (University of Iowa, US)
David Moyes (King’s College London, UK)
Liliane Mukaremera (University of Exeter, UK)
Julian Naglik (King’s College London, UK)
Jim Naismith (Rosalind Franklin Institute, UK)
Jeneil Nett (University of Wisconsin, US)
Deborah O'Neil (Novabiotics, UK)
Phil Packer (Innovate UK, UK)
Peter Pappas (University of Alabama, US)
Tom Patterson (University of Texas, US)
Alessandro Pasqualotto (Universidade Federal de Ciências da Saúde de Porto Alegre, BR)
David Perlin (Center for Discovery and Innovation, Hackensack Meridian Health, US)
Rajendra Prasad (Amity University, IN)
Janet Quinn (Newcastle University, UK)
John Rex (AMR Solutions, US)
Gordon Ramage (University of Glasgow, UK)
Johanna Rhodes (Imperial College, UK)
Angela Riviera (Duke University, US)
P. David Rogers (University of Tennessee, US)
Elizabeth Rowse (Natural Environment Research Council, UK)
Taylor Sandison (Cidara Therapuetics, US)
Dominique Sanglard (Centre Hospitalier Universitaire Vaudois, CH)
Erika Shor (Center for Discovery and Innovation, Hackensack Meridian Health, US)
Emily Stevenson (University of Exeter, UK)
Neil Stone (University College London Hospital, UK)

Jane Usher (University of Exeter, UK)
Adilia Warris (University of Exeter, UK)
Nathan Wiederhold (University of Texas Health Science Center at San Antonio, US)
Lewis White (University Hospital of Wales, UK)
